# Revision of the family Carabodidae (Acari, Oribatida) V. Fourth part. Two new species of the genus *Congocepheus* from the Republic of Rwanda: *Congocepheus rwandensis* sp. n., and *Congocepheus kayoveae* sp. n.

**DOI:** 10.3897/zookeys.556.7011

**Published:** 2016-01-21

**Authors:** Nestor Fernandez, Pieter Theron, Sergio Leiva

**Affiliations:** 1 National Council of Scientific and Technological Research (CONICET). Evolutive Genetic Laboratory FCEQyN, Misiones National University. Felix de Azara 1552, 6º, (3300) Posadas Misiones (Argentina); 2 Research Unit for Environmental Sciences and Management, North-West University, Potchefstroom Campus, 2520 (South Africa); 3 Fellowship, National Institute Agricultural Technology (INTA). Experimental Rural Agency, Aimogasta

**Keywords:** Congocepheus rwandensis sp. n., Congocepheus kayoveae sp. n., Rwanda, comparison

## Abstract

Two new species from Rwanda are described utilizing optical and scanning electron microscope observations: *Congocepheus rwandensis* **sp. n.** and *Congocepheus kayoveae* **sp. n.** are compared to *Congocepheus taurus* Balogh 1961.

## Introduction

Several species of the genus *Congocepheus* have recently been redescribed, namely *Congocepheus heterotrichus* Balogh, 1958, *Congocepheus orientalis* Mahunka, 1987, *Congocepheus hauseri* Mahunka, 1989 (Fernandez et al. 2013c) and *Congocepheus involutus* Mahunka, 1997, with descriptions of new species *Congocepheus gabonensis* Fernandez et al., 2013, *Congocepheus ektactesi* Fernandez et al., 2013 and *Congocepheus germanicus* Fernandez et al., 2014a. A redefinition of *Congocepheus* was given, and the new genus *Cavaecarabodes*, related to *Congocepheus*, was defined and two new species described: *Cavaecarabodes pulchritude* Fernandez et al., 2014a, and *Cavaecarabodes anouchkai* Fernandez et al., 2014a.

*Cavaecarabodes* includes some species previously considered members of the genus *Congocepheus*. Type specimens of *Congocepheus ornatus* Mahunka, 1983, *Congocepheus latilamellatus* Mahunka, 1984 and *Congocepheus velatus* Mahunka, 1986 were studied to establish their position in *Congocepheus*, and at the same time large collections of material were obtained from Rwanda, Zimbabwe, Kenya, Cameroon, the Republic of the Congo, Côte d’Ivoire and Thailand, which are housed at the Natural History Museum of Geneva (NHMG); and from Antilles, Namibia, the Democratic Republic of the Congo, Central African Republic, Tanzania, Ethiopia, Angola, Sudan and South Africa, from the Museum National d’Histoire Naturelle in Paris, France (MNHN). From this material numerous specimens of *Congocepheus* and related genera have been identified. A decision was made to continue with the series of studies of *Congocepheus*, including a number of very interesting new species related to *Congocepheus ornatus*, *Congocepheus latilamellatus* and *Congocepheus velatus*.

In this paper, two new species from Rwanda are described, making use of optical microscopy and SEM. Valuable information was obtained from SEM studies, which would be extremely difficult to obtain with optical microscopy alone.

## Material and methods

Specimens studied by means of light microscopy were macerated in lactic acid and observed in the same medium using the open-mount technique (cavity slide and cover slip) as described by Grandjean (1949) and Krantz and Walter (2009). Drawings were made using a Zeizz GFL (West Germany) compound microscope equipped with a drawing tube.

Specimens were also studied by means of scanning electron microscope (SEM). Specimens preserved in ethanol were carefully rinsed by sucking them several times into a Pasteur pipette, after which they were transferred to buffered glutaraldehyde (2.5%) in Sörensen phosphate buffer: pH 7.4; 0.1 m for two hours. After postfixation for 2 hours in buffered 2% OsO4 solution and being rinsed in buffer solution; all specimens were dehydrated in a series of graded ethanol and dried in a critical point apparatus. After mounting on Aluminium-stubs with double sided sticky tape, specimens were gold coated in a sputter apparatus (Alberti and Fernandez 1988, 1990a, 1990b; Alberti et al. 1991, 1997, 2007; Fernandez et al. 1991). SEM observations were made using a FEI-Quanta Feg 250; with 10 Kv and working distance (WD) variable. Measurements taken: total length (tip of rostrum to posterior edge of notogaster); width (widest part of notogaster) in micrometres (μm).

Leg chaetotaxy studies using standard, polarized and phase contrast microscopes are provisional, due to the fact that only adult specimens were available. Setal formulae of the legs include the number of solenidia (in parentheses); tarsal setal formulae include the famulus (ε).

### Morphological terminology and abbreviations

Morphological terms and abbreviations used are those developed by F. Grandjean (1928–1974) (cf. Travé and Vachon 1975; Norton & Behan-Pelletier (in Krantz and Walter 2009); Fernandez et al. 2013; 2013c; 2014). For setal types Evans 1992: 73; and for ornamentation of cuticular surfaces Murley 1951(in Evans *op.cit*: 9) were used.

## New taxa descriptions

### Congocepheus rwandensis sp. n.

Taxon classificationAnimaliaOribatidaCarabodidae

http://zoobank.org/6675DD70-7300-426B-A766-0141BFE4C766

Figures 1–3 , 4–6 , 7–10 , 11–14 , 15–18 , Table 1

#### Etymology.

The specific epithet is derived from Rwanda, country of origin of the type material.

#### Material examined.

Holotype: Female. “73/2. Kayove-Rwanda; 2100 mts.15/V/1973” Leg. P.Werner; material deposited in the Collection of the Natural History Museum of Geneva (MHNG), Switzerland; preserved in 70% ethanol.

Paratype: two adult females, same locality and date as Holotype; deposited in Collection of MHNG; preserved in 70 % ethanol. Material studied for SEM: three specimens, not deposited.

#### Diagnosis adult female.

Integumental microsculpture: prodorsal, notogastral and ventral zones smooth to slightly irregularly tuberculate; notogaster with irregular cord-shaped structures and short, fingerlike projection. Setation: with medial dentate vein: rostral setae small; interlamellar setae large, directing backward; lamellar: wide, short with central dentate vein; notogastral, epimeral, genital, aggenital, anal and adanal setae: simple. Prodorsum: wide elevated interlamellar process; *in* setae anteriorly on elevated zone; sensillus: cylindrical, short barbs. Bothridial ring, bothridial tooth present, smooth. Large posterior prodorsal depression. Notogaster: small notogastral anterior depression; fourteen pairs of setae. Lateral zone: tutorium: large lamina, small relative to pedotectum I. Ventral region: epimera slightly elevated; 3-4 fused; epimeral chaetotaxy 3-1-3-3; discidum clearly discernible; anterior genital furrow clearly visible; four pairs of genital setae in a unique line; aggenital setae inserted posterior to genital opening. Three pairs of adanal seta; *ad_3_* near aggenital setae; anal plate polyhedral, sharply tipped; two pairs of anal setae; lyrifissures *iad* between *ad_3_* and *ad_2_*; conspicuous depressions situated laterally to genital and anal openings.

#### Description.

***Measurements*.** SEM: 475 μm (473–477) × 225 μm (223–227) (measurements on three specimens). Light microscopy: 476 μm (473–478) × 227 μm (228–227) (measurements on three specimens).

***Shape*.** Elongate oval (Figures 4, 7).

**Figures 1–3.**
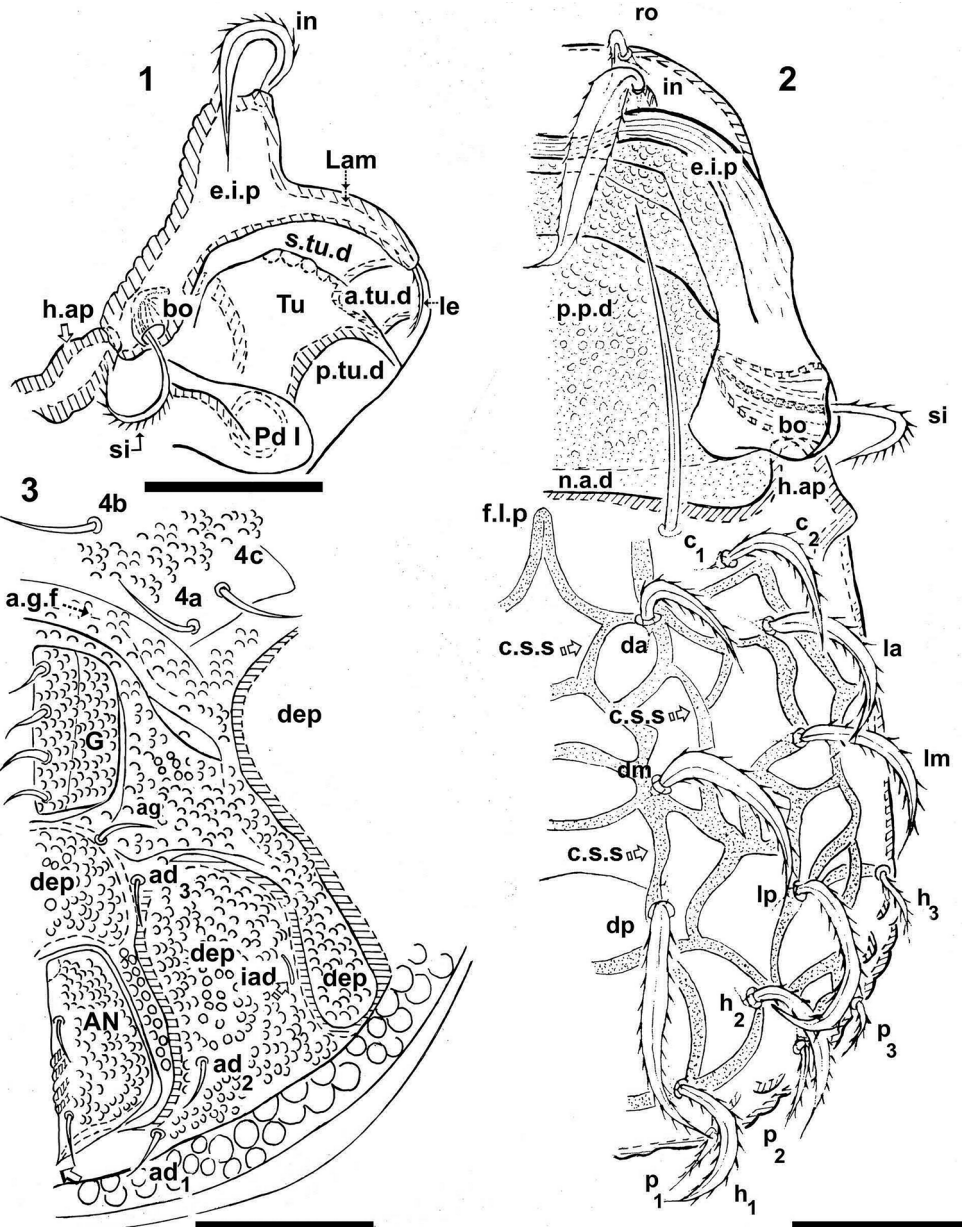
*Congocepheus rwandensis* sp. n. Adult female, optical observations. **1** prodorsal zone, anterolateral view **2** partial ventral view **3** dorsal view. Abbreviations: see “Material and methods”. Scale bars: (**1, 3**) 100 μm; (**2**) 85 μm.

**Figures 4–6.**
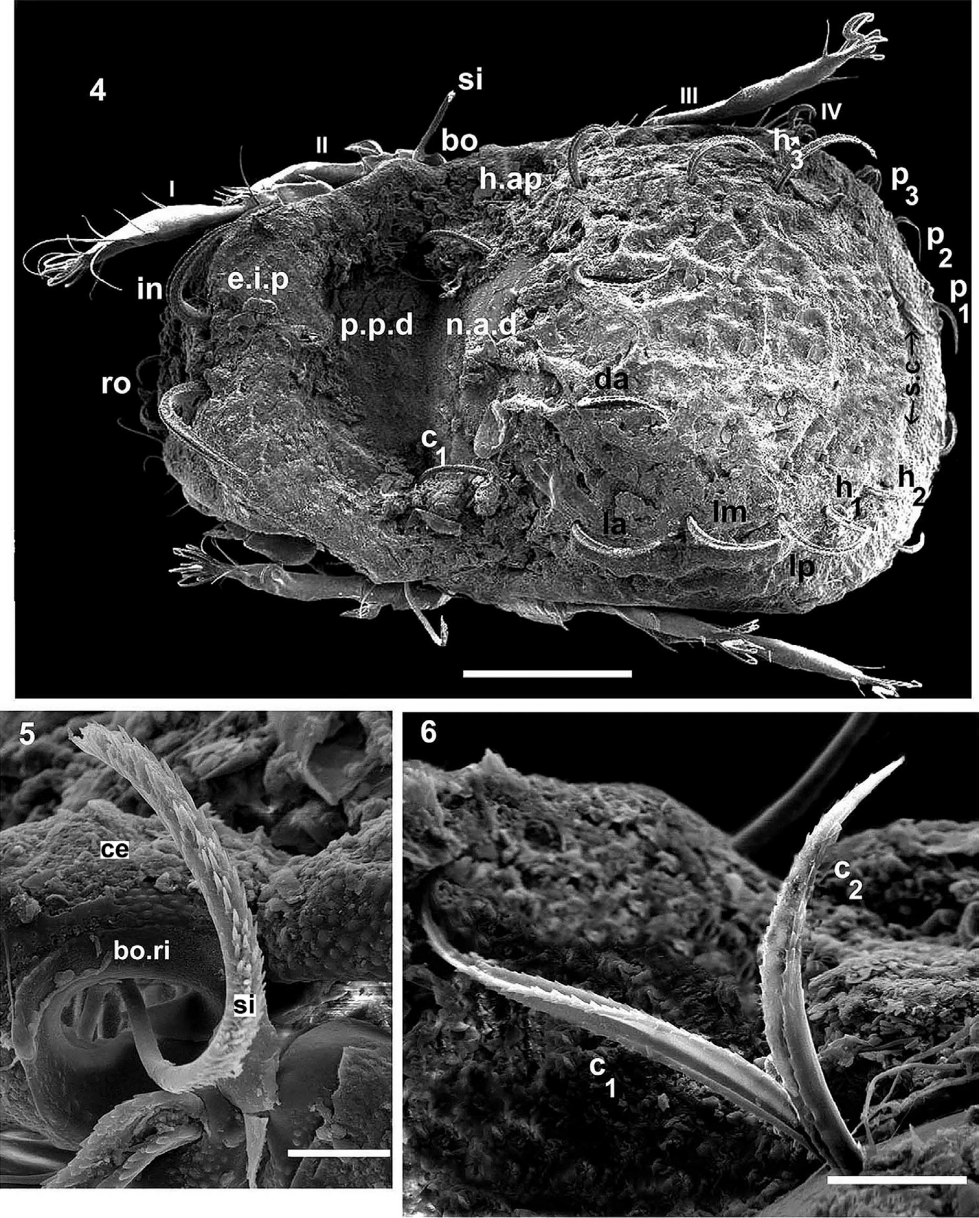
*Congocepheus rwandensis* sp. n. Adult female, SEM. **4** dorsal view **5** lateral view of bothridial ring and sensillus **6** lateral view *c_1_*, *c_2_* setae. Abbreviations: see “Material and methods”. Scale bars: (**4**) 100 μm; (**5**) 10 μm; (**6**) 20 μm.

***Colour*.** Specimens without cerotegument, light brown to yellowish-brown when observed in reflected light.

**Figures 7–10.**
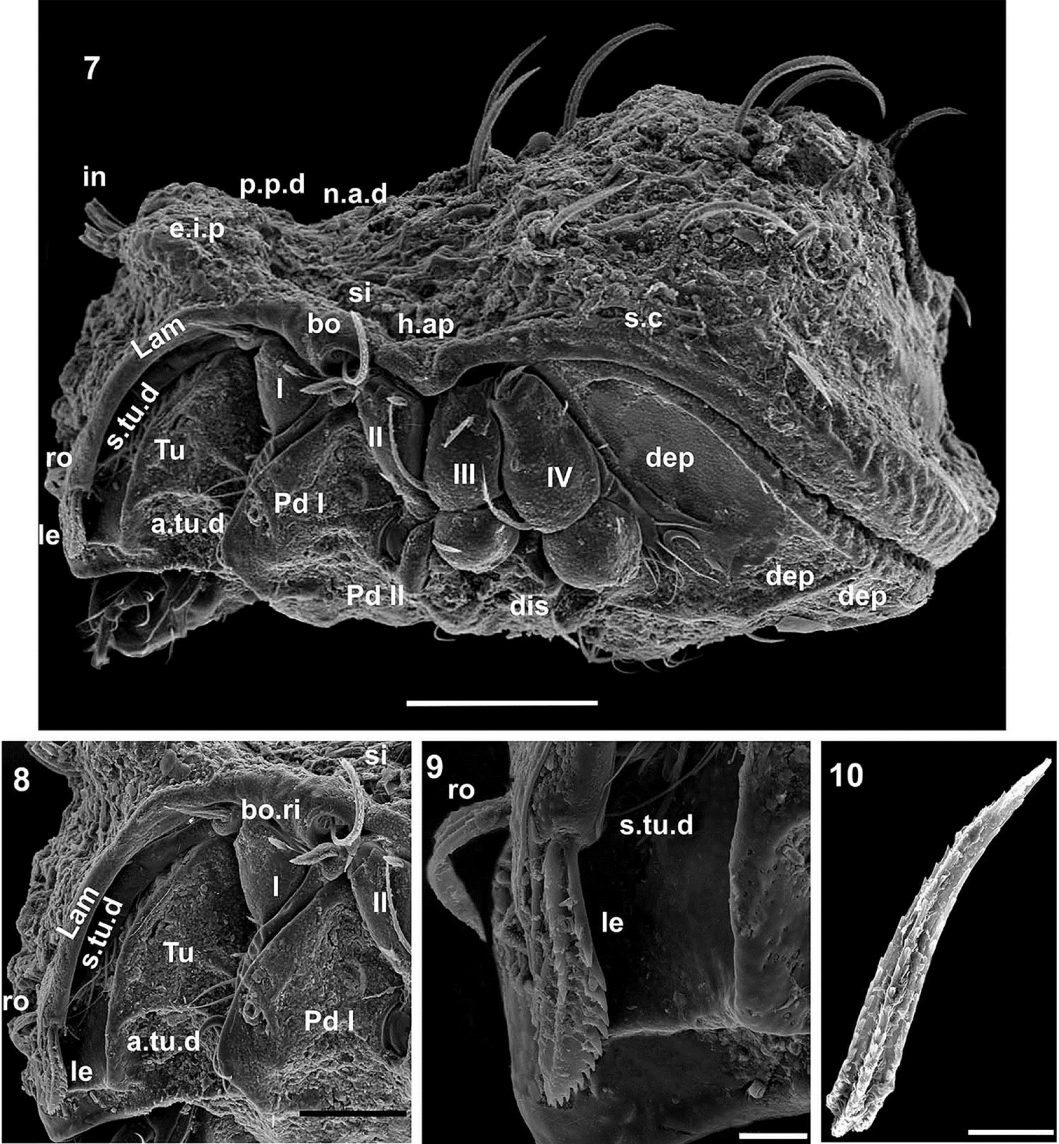
*Congocepheus rwandensis* sp. n. Adult female, SEM. **7** lateral view, without *c_1_*, *c_2_* setae **8** anterior lateral zone **9** lamellar setae **10** notogastral setae. Abbreviations: see “Material and methods”. Scale bars: (**7**) 100 μm; (**8**) 50 μm; (**9, 10**) 10 μm.

***Cerotegument*.** Present on: prodorsum, notogaster, ventral region. Consistently granulated to amorphous layer covering body (1.5–3.0 μm), with adhering soil particles, impeding observation of cuticular ornamentations; on legs thin layer (less than 1 μm) (Figures 4, 6, 7).

Absent on: lamellar lateral border (*Lam*), bothridial ring (*bo.ri*), humeral apophysis (*h.ap*) and bothridium (*bo*) (Figures 5, 7, 8).

Sometimes absent: ventral depression (*dep*) behind leg IV and notogastral zone between *s.c* and notogastral edge (Figure 7).

**Figures 11–14.**
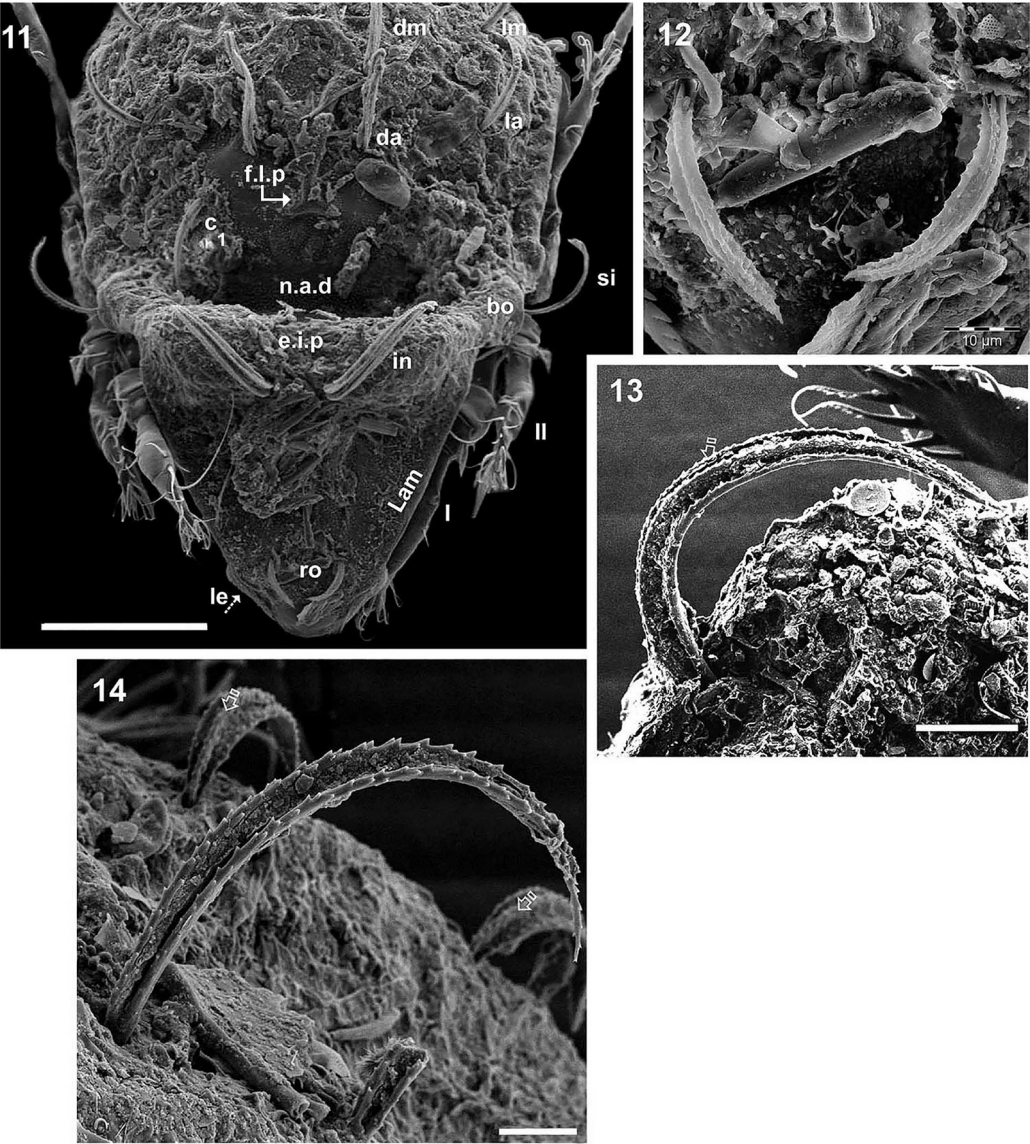
*Congocepheus rwandensis* sp. n. Adult female, SEM. **11** frontal view **12** rostral setae **13** interamellar setae **14** notogastral setae. Abbreviations: see “Material and methods”. Scale bars: (**11**) 100 μm; (**12, 14**) 10 μm; (13) 20 μm.

***Integument*.** Microsculpture: *smooth* to *slightly irregular tuberculate* (Figures 2, 3, 5, 11): prodorsal, notogastral and ventral zones. Lateral zone of notogaster presenting slightly larger tubercles. Fingerlike projection (*f.l.p*) clearly visible on central notogastral zone (Figures 2, 11).

**Figures 15–18.**
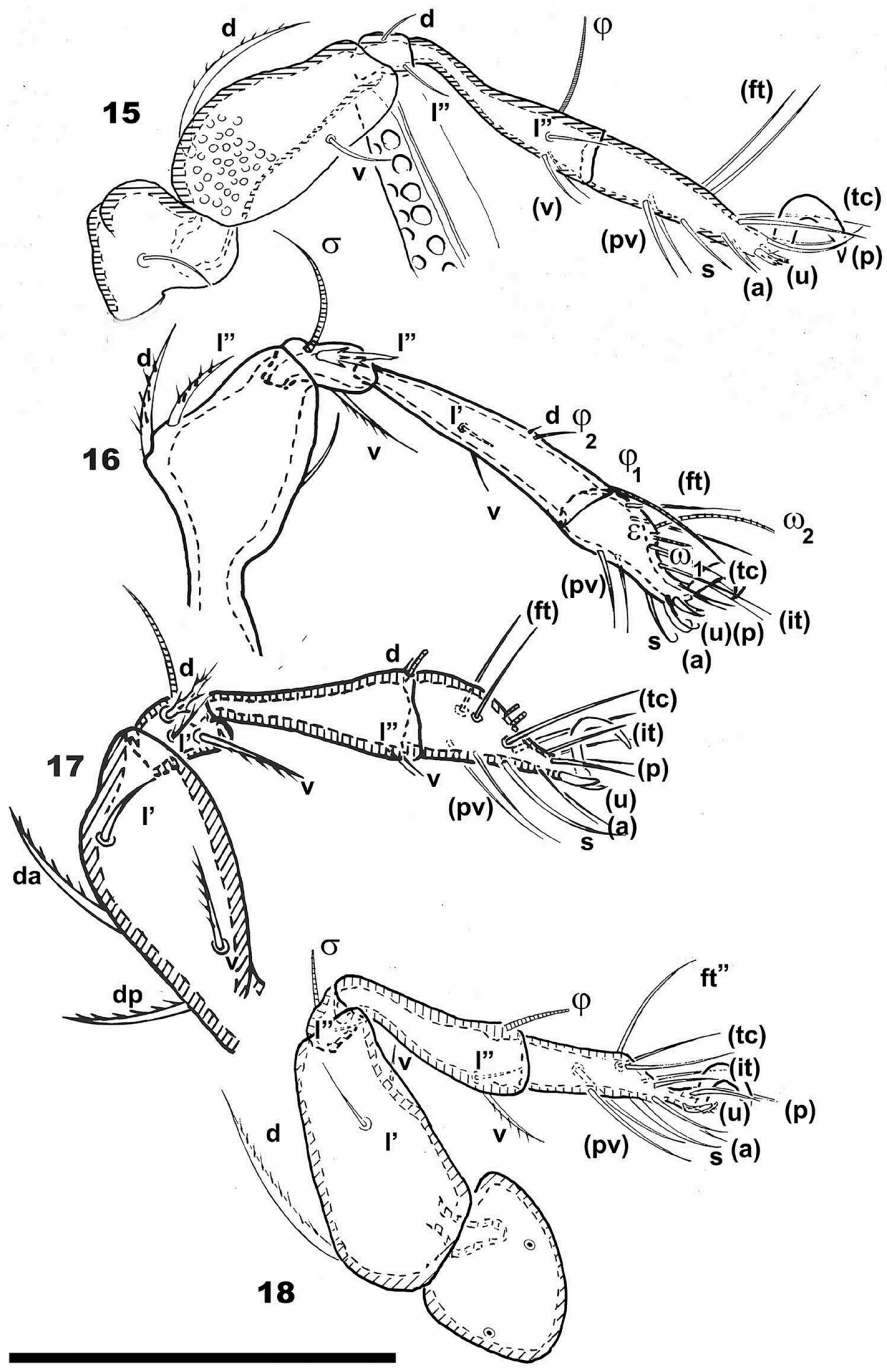
*Congocepheus rwandensis* sp. n. Adult female, optical observations. **15** leg IV, antiaxial **16** leg I, antiaxial **17** leg II, antiaxial **18** leg III, antiaxial. Abbreviations: see “Material and methods”. Scale bar: (**15–18**) 130 μm.

On central notogastral zone, network of irregular cord-shaped structures (*c.s.s*) (indicated byX, Figure 2) extending to setal insertion zone, *c.s.s* terminating in fingerlike projection (*f.l.p*) on anterior notogastral zone.

***Setation*.** SEM-observations were necessary in order to determine setal shapes. Notogastral and prodorsal setae (*ro*, *in*) (Figures 1, 2, 6, 10, 11, 12, 13, 14) with elevated medial vein and dentitions; lateral setal margin dentate (Figure 10); *ro* setae small (Figures 11, 12); *in* large directing backwards (Figures 1, 4). In many cases the setae are twisted, immensely complicating observation; the presence of small particles adhering to setal surfaces, further obscuring observation.

Wide, short *le* setae (Figure 9) with central vein and dentitions. Notogastral setae with medial dentate veins and dentate margin (Figures 10, 14 central vein indicated by X). Epimeral, genital, aggenital, anal and adanal setae simple, sharply tipped (Figure 3).

***Prodorsum*.** Polyhedral (dorsal view) (Figure 2, 4); convex polyhedral in lateral view (Figures 1, 7); triangular in frontal view (Figure 11). Elevated wide interlamellar process (*e.i.p*) (Figures 1, 4, 7, 11); *e.i.p* complete, with small depression in medial zone. Anteriorly situated setae *in* on elevated zone of *e.i.p*; *in* setae large (70 ± 5 μm), initially directing forward but tips curving backward; *in* setae inserted antiaxially to medial plane and slightly internally to *ro* insertion level (Figures 1, 2, 11, 13). Clearly visible *ro* setae, length 33 ± 3 μm, curving towards medial zone, apical tips adjacent to each other (Figures 9, 11, 12); *le* setae lateral, length 26 ± 3 μm and 12 ± 3 μm in the wider zone (Figure 9); *ro* setal insertion at level of *le* setal insertion.

Sensillus (*si*) cylindrical with short barbs (Figure 5). Bothridial ring (*bo.ri*) smooth, well defined, with bothridial tooth (Figure 5). Posterior prodorsal depressed zone (*p.p.d*) conspicuous, with notogastral anterior depression (*n.a.d*) (Figures 2, 4, 7) delimiting a large depressed area. Rostral margin slightly rounded to hexagonal (Figure 11). Lamellae lateral; lamellar tip not observed, shallow lamellar furrow not discernible.

***Notogaster*.** Shape: in dorsal view anterior rectangular, in posterior view oval (Figure 2, 4); in lateral view anterior clearly depressed and rest convex (Figure 7); *d.sj* narrow, slightly rectilinear, well delimited; notogastral anterior depression (*n.a.d*) small (Figures 2, 4).

Fourteen pairs of setae: *c*_1_, *c*_2_, *da*, *dm*, *dp*, *la*, *lm*, *lp*, *h*_1_, *h*_2_, *h*_3_, *p*_1_, *p*_2_, *p*_3_; *c*_1_ setae directing forward (Figures 2, 6), other setae directing backward (Figures 2, 4, 7), *c*_1_ largest; *h*_3_, *p*_1_, *p*_2_, *p*_3_ smaller; c_2_, *da*, *dm*, *dp*, *la*, *lm*, *la*, *h*_1_, *h*_2_ more or less equal in length. Series of irregular *c.s.s* in central zone converging to form a short *f.l.p* (Figure 2). Circumgastric depression (*s.c*) situated in front of *p*_1_, *p*_2_, *p*_3_, *h*_3_ setae (Figure 4) clearly visible in posterior notogastral area). Humeral apophysis (*h.ap*) very long, clearly visible as large elongate projection resulting in characteristic shape of anterior notogastral zone (Figure 4).

***Lateral region*** (Figure 7). Lamellae (*lam*) well discernible, more or less truncate; cuticular surface of lamellar zone smooth, always without cerotegumental layer. Tutorium (*tu*) a prominent curving lamina, margin clearly discernible, smooth cuticula.

Deep supra tutorial depression (*s.tu.d*) running between and parallel to lamellae and tutorium; large pocket depression (*a.tu.d*) anteriorly. Pedotectum I, large extended lamina, covering acetabulum I, rounded apex. Pedotectum II, small ovoid lamina; discidium (*dis*) well discernible, small, triangular, rounded apex.

Bothridia cup-shaped; bothridial opening directing downward (Figures 5, 7); smooth bothridial ring (*bo.ri*) wider in inferior zone, *bo.ri* incomplete with bothridial tooth, clearly discernible. Sensilllus cylindrical with barbs arching toward the tip (Figures 5, 7). Humeral apophysis (*h.ap*): elongate extended structure, rounded apex, basally curved; anterior tip overlapping posterior bothridial part. Clearly visible large depression (*dep*) behind leg IV; two other *dep* present in lateral and posterior anal zones.

***Ventral region*** (Figure 3). Epimera slightly elevated, delimited by a narrow but deep furrow (*bo.1*, *bo.2*, *bo.sj*). Epimera 4 fused, epimeral furrow (*bo.3*) narrow; *apo.1*, *apo.2*, *apo.sj* and *apo.3* well discernible.

Epimeral chaetotaxy 3-1-3-3. Discidum easily discernible; anterior genital furrow (*a.g.f*) clearly visible, situated in front of genital plate. Large genital plate; four pairs of genital setae, simple linear arrangement; all setae more or less equal in length; aggenital setae (*ag*) situated posteriorly to genital opening. Three pairs of adanal seta; *ad_3_* close to *ag* setae. Anal plate polyhedral, sharply tipped. Two pairs of anal setae. Lyrifissures *iad* well discernible, situated laterally between *ad_3_* and *ad_2_*. Depressions (*dep*) clearly visible, situated laterally to genital and anal openings.

***Legs*** (Figures 9–12). All legs monodactyle. Setal formulae I (1-3-2-3-16-1) (1-2-2); II (1-4-3-3-15-1) (1-1-2); III (2-3-1-2-14-1) (1-1-0); IV (1-2-2-3-13-1) (0-1-0). See Table 1.

**Table 1.** *Congocepheus rwandensis* sp. n. setae and solenidia.

Leg I	Femur	Genu	Tibia	Tarsus	Claw
setae	*d*,*l*”,*v*	*l*”,*v*	*v*,*l*’,*d*	(*ft*),*ε*,(*tc*),(*it*),(*p*), (*u*),(*a*),*s*,(*pv*)	1
solenidia		σ	φ_1_, φ_2_	ω _1_, ω _2_	
**Leg II**					
setae	*dp*,*da*,*l*,*v*	*d*,*l*’,*v*	*v*,*d*,*l*”	(*pv*),*s*,(*a*),(*u*),(*p*), (*it*),(*tc*),(*ft*)	1
solenidia		*σ*	*φ*	ω _1_, ω _2_	
**Leg III**					
setae	*d*,*l*’,*v*	*l*’’	*l*”,*v*	(*pv*),*s*,(*a*),(*u*),(*p*), (*it*),(*tc*),*ft*’’	1
solenidia		σ	φ	-	
**Leg IV**					
setae	*d*,*v*	*d*,*l*’’	*l*”(*v*)	(*pv*),*s*,(*a*),(*u*),(*p*), (*tc*),(*ft*)	1
solenidia		-	φ	-	

#### Remarks.

The cerotegumental layer impedes clear observation of *c.s.s* and *f.l.p*. Observation of notogastral setae was complicated due their length and the fact that they are twisted. Residues adhering to setal surfaces further hampered clear observation.

### Congocepheus kayoveae sp. n.

Taxon classificationAnimaliaOribatidaCarabodidae

http://zoobank.org/BFD45F16-4D42-4684-81C2-17A3D5F723C7

Figures 19–24 , 25–28 , 29–33 , 34–38 , 39–43 , 44–47 ; Table 2

#### Etymology.

The specific epithet is derived from Kayove, Rwanda, where the type material was collected.

#### Material examined.

Holotype Female. “73/2. Kayove- Rwanda; 2100 mts. 15/V/1973” Leg. P.Werner; material deposited in the Collection of the Natural History Museum of Geneva (MHNG), Switzerland; preserved in 70% ethanol. Four adult female paratypes, same locality and date as holotype; deposited in Collection of MHNG; preserved in 70 % ethanol. Material studied by SEM: six specimens, not deposited.

#### Diagnosis adult female.

Integumental microsculpture: notogaster with irregular cord-shaped structures and elongate fingerlike projection.

Setation: *simple*: epimeral, genital, aggenital, anal, adanal, subcapitular; *one central dentate vein, margin dentate*: notogastral; *two dentate veins, margin dentate*: rostral, interlamellar; *flat setae, margin dentate, central dentate vein*: lamellar.

Prodorsum: elevated interlamellar process complete; margin of laterodorsal lamellae slightly elevated. Prominent triangular lamellar tip, lamellar setae situated externally; shallow lamellar furrow terminating near internal limit of lamellar tip. Tutorium spoon-shaped, larger than Pedotectum I. Rostrum: rounded undulate margin, wide, large, projecting forward. Epimera elevated, delimited by deep furrow; deep hollow paraxially to epimere 1; epimeres 3 and 4 unfused. Epimeral chaetotaxy 3-1-3-3. Genital plate rounded; four or five pairs of genital setae; aggenital setae posterior to genital opening, far from *ad_3_*.

#### Description.

***Measurements*.** SEM: 464 μm (462–467) × 173 μm (172–180) (measurements on six specimens). Light microscopy: 467 μm (465–468) × 175 μm (173–183) (measurements on five specimens).

**Figures 19–24.**
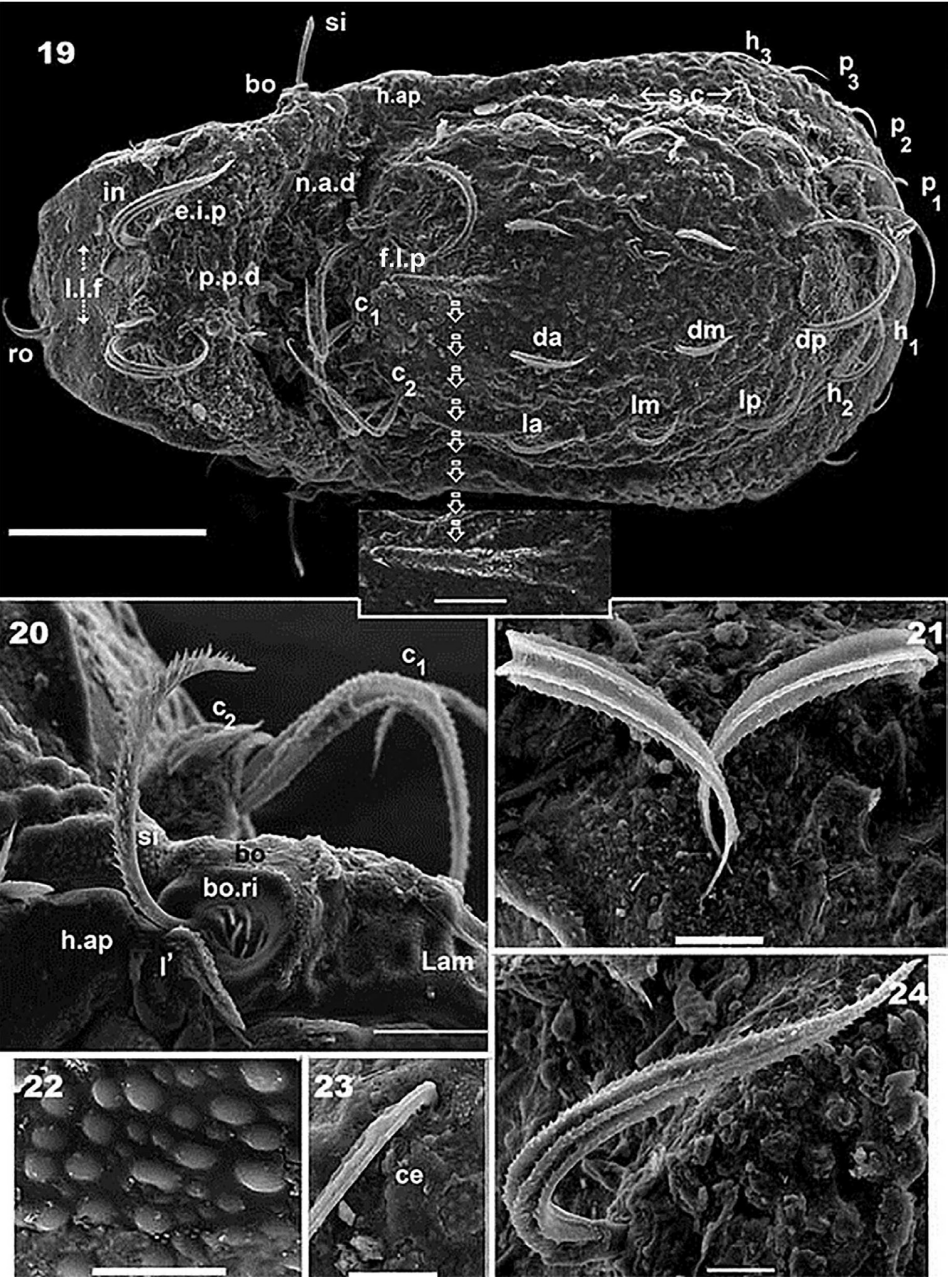
*Congocepheus kayoveae* sp. n. Adult female, SEM. **19** dorsal view, with detail of fingerlike projection (*f.l.p*) **20** lateral view, bothridium, sensillus and humeral apophysis **21** rostral setae **22** cuticular microsculpture **23** cerotegumental layer and cuticular microsculpture **24** interlamellar setae. Abbreviations: see “Material and methods”. Scale bars: (**19**) 100 μm (detail of *f.l.p* = 20 μm); (**20**) 20 μm; (**21, 23, 24**) 10 μm; (**22**) 5 μm.

***Shape*.** Elongate oval (Figure 19). ***Colour*.** Specimens without cerotegument; light brown to yellowish-brown when observed in reflected light.

***Cerotegument*.** Present: thin amorphous layer (0,3–0,5 μm) on prodorsum, notogaster, ventral region; with adhering soil particles principally on *e.i.p* and central notogastral zone (Figures 19, 21, 22, 23, 24). Observation of cuticular ornamentation not impeded by cerotegumental layer (Figures 20, 22, 29, 31). Absent: bothridial ring (*bo.ri*) (Figure 20).

**Figures 25–28.**
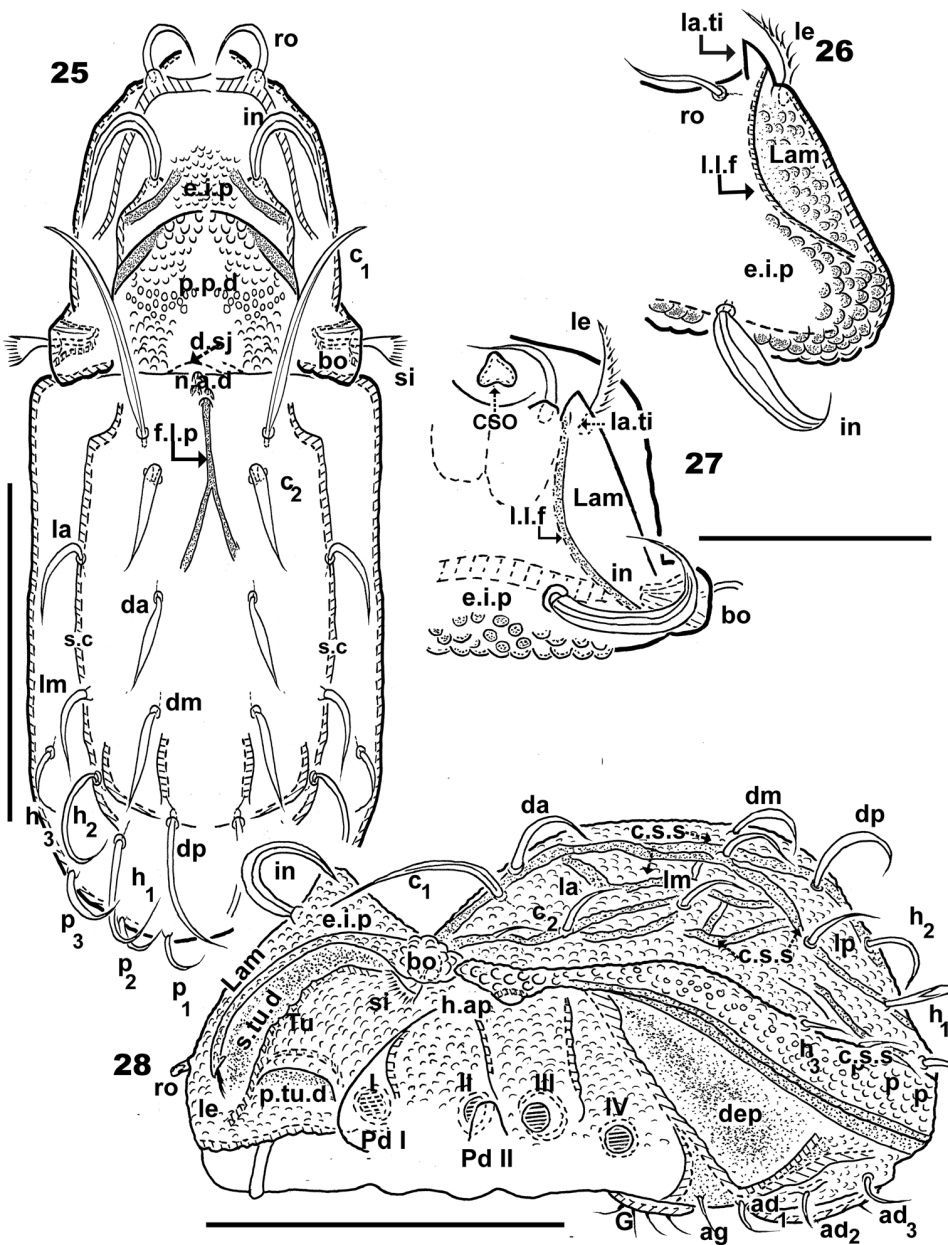
*Congocepheus kayoveae* sp. n. Adult female, optical observations. **25** dorsal view **26** prodorsum, frontal inclined view **27** prodorsum, dorsal inclined view **28** lateral view. Abbreviations: see “Material and methods”. Scale bars: (**25, 28**) 220 μm; (**26, 27**) 80 μm.

**Figures 29–33.**
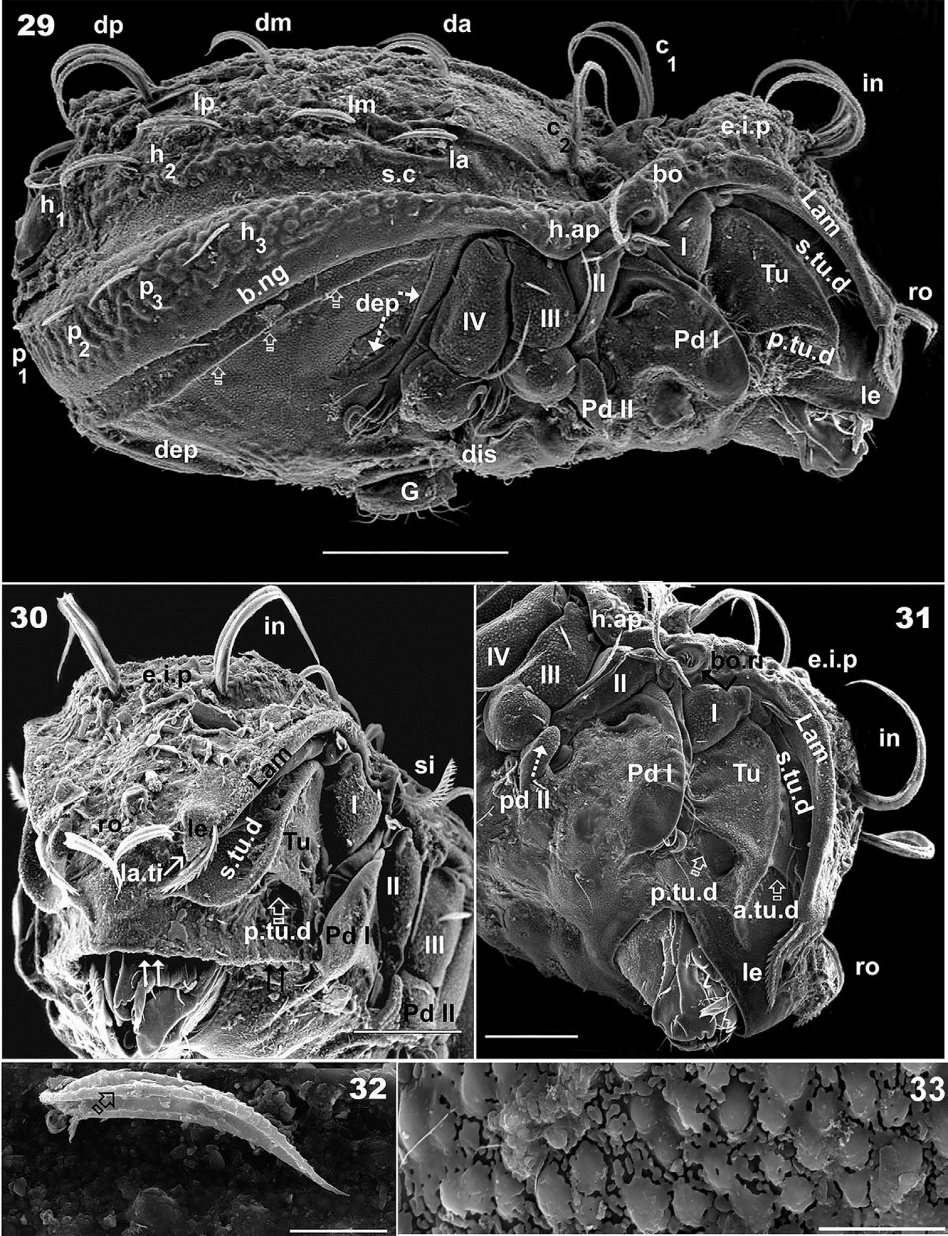
*Congocepheus kayoveae* sp. n. Adult female, SEM. **29** lateral view **30** frontal inclined view **31** lateral inclined view **32** notogastral setae **33** damaged cerotegumental layer. Abbreviations: see “Material and methods”. Scale bars: (**29**) 100 μm; (**30–31**) 50 μm; (**32**) 10 μm; (**33**) 5 μm.

**Figures 34–38.**
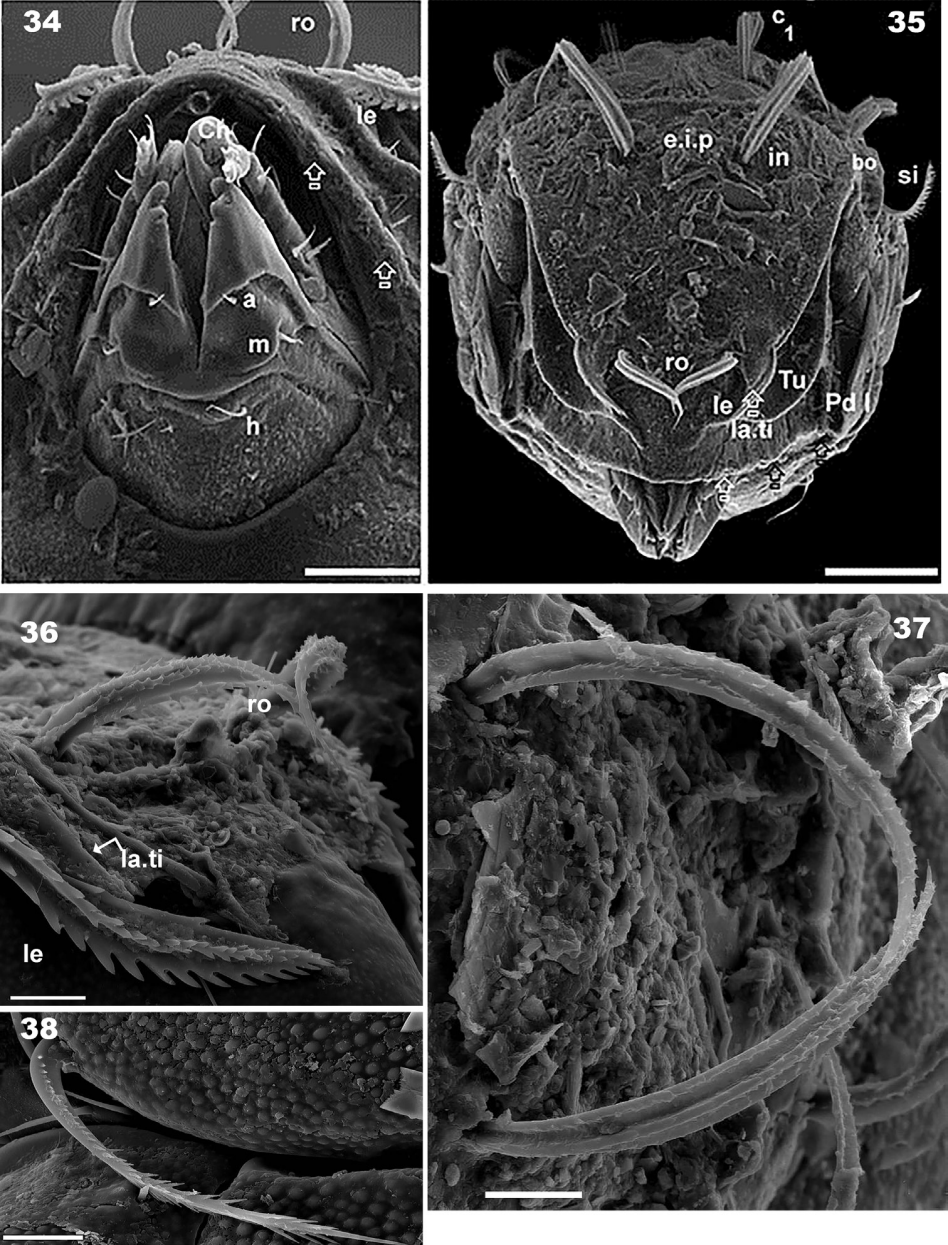
*Congocepheus kayoveae* sp. n. Adult female SEM. **34** subcapitulum, ventral view **35** prodorsum, frontal view **36** lamellar setae (*le*), lateral view **37** setae *dp*, dorsal view **38** seta *d* femur III, lateral view. Abbreviations: see “Material and methods”. Scale bars: (**33**) 20 μm; (**34**) 50 μm; (**35–37**) 10 μm.

**Figures 39–43.**
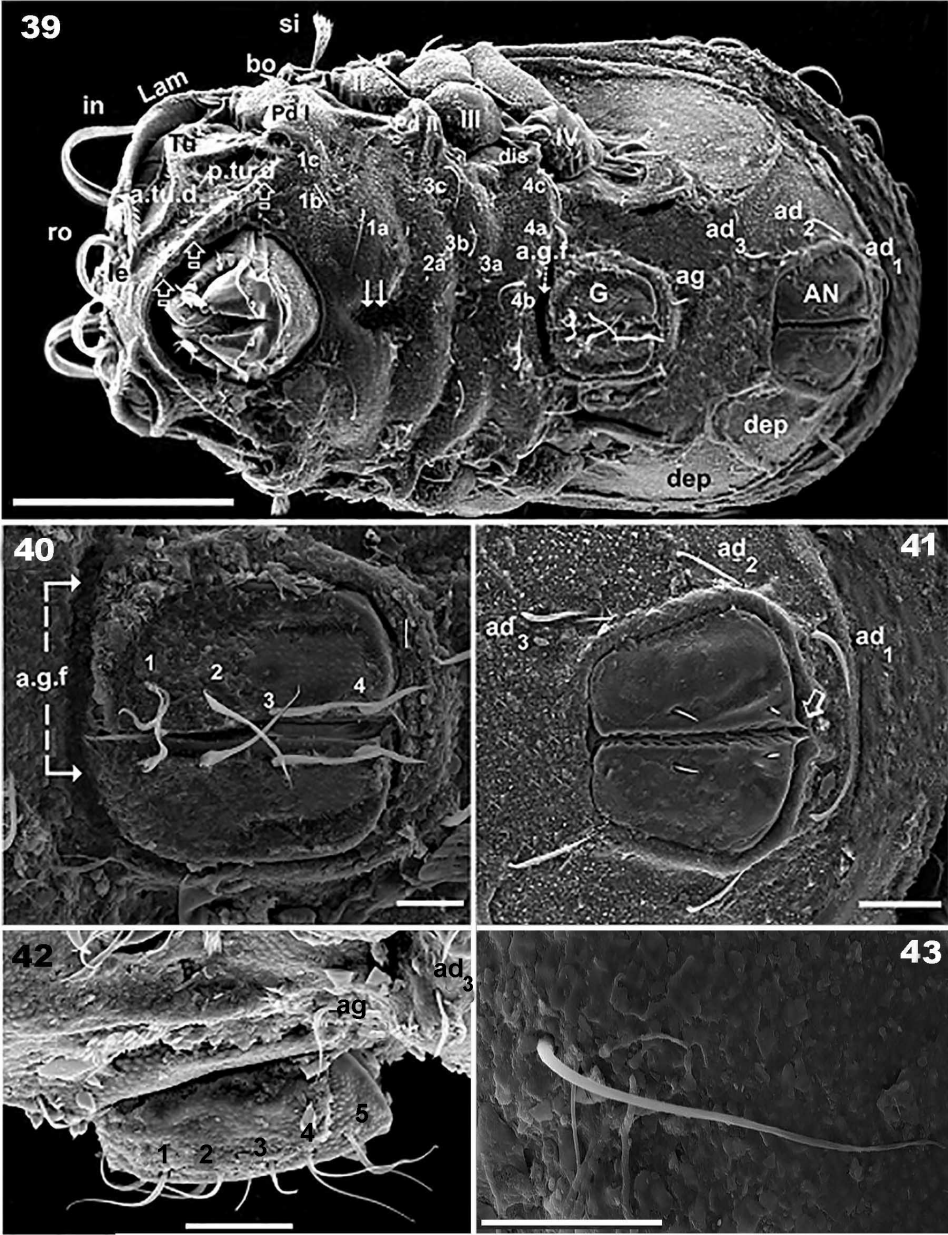
*Congocepheus kayoveae* sp. n. Adult female SEM. **39** ventral view **40** genital plate **41** anal zone **42** genital plate, lateral view **43** epimeral setae. Abbreviations: see “Material and methods”. Scale bars: (**39**) 100 μm; (**40, 42, 43, 44**) 10 μm; (**38, 41**) 20 μm.

***Integument*.** *Pusticulate* (Figure 22): prodorsum: *e.i.p* posterior zone, lamellar margin and bothridial zone; central notogastral zone and humeral apophysis (Figures 19, 20, 29); legs: femurs (Figure 38). Smooth to granulate: prodorsum: anterior *e.i.p* (Figure 19); notogaster: *s.c*, *b.ng* zone (Figure 29); lateral zone: *Tu*, *s.tu.d*, *Pd I*, *Pd II*. Ventral zone: subcapitulum, epimeral, genital, anal and *dep* (Figures 29, 30, 31, 34, 39, 40, 41, 42). Series of irregular *c.s.s.* on notogastral zone, forming central elongate *f.l.p* (Figures 25, 28) (described in detail under notogaster), well visible without cerotegumental layer.

**Figures 44–47.**
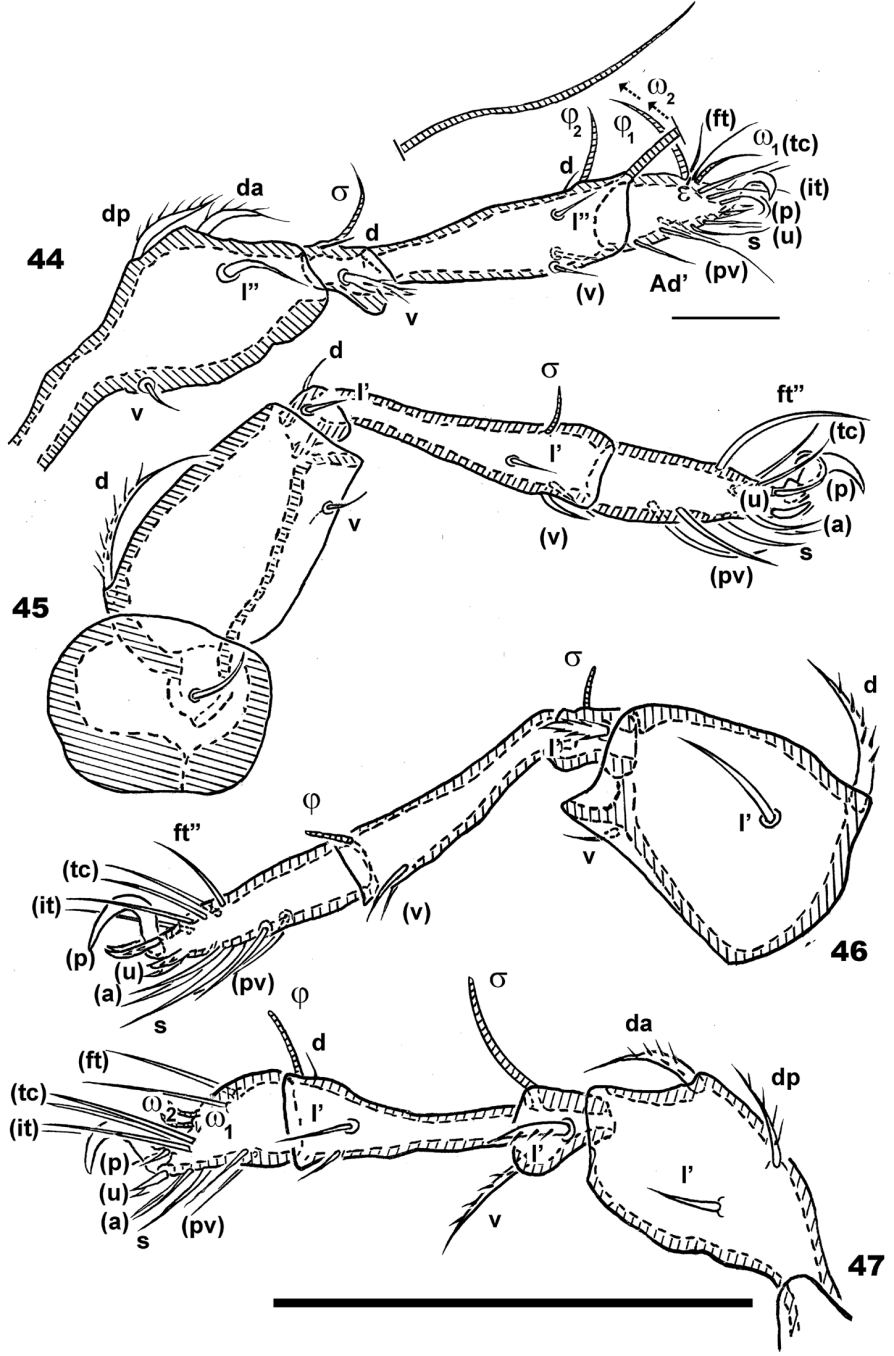
*Congocepheus kayoveae* sp. n. Adult female, optical observations. **44** leg I antiaxial **45** leg IV, antiaxial **46** leg III, antiaxial **47** leg II antiaxial. Abbreviations: see “Material and methods”. Scale bar: (**44–47**) 100 μm.

***Setation*.** *Simple*: epimeral (Figures 39, 43), genital (Figures 40, 42), aggenital (Figure 42), anal, adanal (Figure 41), subcapitular (Figure 34) and seta of genu, tibia and tarse of legs. *One central dentate vein and dentate margin*: notogastral (Figures 32, 37); two types of notogastral setae: large *c_1_*, *c_2_*, *dp*, *h_1_*, with upward directing dentate margin (Figure 37); small *da*, *dm*, *la*, *lm*, *lp*, *h_2_*, *h_3_*, *p_1_*, *p_2_*, *p_3_*, dentate margin not directing upward (Figure 32). *Two dentate veins and dentate margin* (Figures 21, 24), *ro* (Figure 21), *in* setae (Figure 24). *Flat setae, dentate margin with central dentate vein*: *le* setae (Figure 36), superior margin presenting few teeth; numerous large teeth on inferior margin. *Barbate, with central dentition*: large femoral setae (legs I-IV) (Figure 38).

***Prodorsum*.** Polyhedral (dorsal view) (Figure 19); slightly convex polyhedral in lateral view (Figure 29); triangular in frontal view (Figure 35). Elevated interlamellar process (*e.i.p.*) almost flat in dorsal view (Figures 19, 25); large surface between *p.p.d* and *in* setae insertion zone (Figures 19, 29, 35), flat in frontal view (Figure 35) (see Remarks); *in* setae (Figure 24) large (82 μm ± 5), curving, directing backward (Figures 19, 29, 30, 31, 35), setae inserted anteriorly on *e.i.p.* (Figures 29, 30, 31) at same longitudinal level as *ro* insertion (Figure 35); *ro* setae (Figure 21) length (38 μm ±3 μm), directing forward and paraxially (Figures 29, 30), curving downward (Figures 29, 36) with criss-crossing tips (Figures 21, 30, 35); *le* setae lateral (Figures 29, 35, 36), length (58 μm ± 3), wider zone (10 μm ± 2); *ro* and *le* setal insertion at same level.

Sensillus (*si*) (Figure 20) (66 μm ± 3) uncinate, curving upward (Figures 29, 30, 31, 35). Bothridial ring (*bo.ri*) smooth, well defined, with bothridial tooth (Figure 20).

Rostral margin slightly rounded, margin undulate (Figure 30 indicated byJ; 35 indicated by Y).

Lamellae running dorsolaterally (Figures 26, 27); semicircular shallow lamellar furrow (*l.l.f*) originating on bothridial zone and terminating near lamellar tip (*la.ti*), clearly discernible when cerotegumental layer absent (Figures 26, 27). Posterior prodorsal depressed zone (*p.p.d*) large, normal (Figures 19, 25).

***Notogaster*.** Ovoid in dorsal view, with slight constriction at level of *da*, *la* setae (Figure 19); in lateral view zone anterior to *da*, *la* setae slightly depressed, rest convex (Figure 29); *d.sj* narrow, slightly rectilinear, well delimited (Figure 25); notogastral anterior depression (*n.a.d*) reduced (Figures 19, 25).

Fourteen pairs of setae: *c*_1_, *c*_2_, *da*, *dm*, *dp*, *la*, *lm*, *lp*, *h*_1_, *h*_2_, *h*_3_, *p*_1_, *p*_2_, *p*_3_; *c*_1_ (86 ± 5 μm); c_2_ (75 ± 5 μm) both setae long and thin, direction variable but in most cases directing forward (Figure 19, 31, 35), however not uncommon for these setae to be directing backward (Figure 29). Setae *da* (35 ±3 μm); *dm* (30 ± 3 μm); *dp* (65 ± 3 μm); *la* (35 ± 3 μm); *lm* (27 ± 3 μm); *lp* (42 ± 3 μm); *h*_1_ (48 ± 3 μm); *h*_2_ (45 ± 3 μm); *h*_3_ (25 ± 3 μm); *p*_1_ (13 ± 3 μm); *p*_2_ (15 ± 3 μm); *p*_3_ (17 ± 3 μm).

Cord-shaped structures (*c.s.s*) converging in central anterior zone forming an elongate fingerlike projection (*f.l.p*) (53 ± 5 μm (Figure 25). Circumgastric depression (*s.c*) present, clearly visible (Figures 19, 29), from *h.ap* surrounding notogaster, situated between *la*, *lm* , *lp*, *h_2_*, *h_1_* and *h_3_*, *p_3_*, *p_2_*, *p_1_* setae (Figure 19). Humeral apophysis (*h.ap*) large elongate projection (Figure 19).

***Lateral region*** (Figures 29, 31). Lamellae (*lam*) easily discernible; cuticular microsculpture near bothridial zone pusticulate with several round depressions (Figure 20); *le* setal insertion at same level as *ro* setal insertion; conspicuous *la.ti* (Figures 25, 26, 35) (details in frontal view).

Tutorium (*tu*) prominent lamina, curving margin, clearly discernible, smooth cuticula (Figures 29, 31). Deep supratutorial depression (*s.tu.d*) running parallel to and between lamellae and tutorium; *p.tu.d* and *a,tu d* present, large (Figure 31). *Tu* larger than *Pd I*, expanded laterally (Figure 31).

Bothridial ring (*bo.ri*) smooth, with *bo.to*, hardly discernible due to positioning of lateral antiaxial setae (*l*”) of genu II (Figure 20) (see Remarks); *h.ap* triangular, inferior margin rounded; anterior zone of *h.ap* overlapping posterior bothridial zone (Figures 20, 29).

Clearly delimited zone on *s.c* with more or less smooth cuticula, immediately followed by clearly delimited pusticulate zone (Figure 29), and slightly below insertion of setae *h_3_*, *p_3_*, *p_2_*, *p_1_* to *h.ap*, a smooth zone extending to *b.ng*. Clearly delimited depressed zone behind leg IV. Cuticular ribbon (Figure 29 indicated by X) parallel to *b.ng*.

***Frontal view*** (Figures 26, 27, 30, 35). Actual shape and disposition of: *e.i.p*, *in* setae, *Lam*, *le* setae, *la.ti*, *Tu*, *Pd I*, *s.tu.d.* and characteristics of rostral margin visible in frontal view.

Complete, flat *e.i.p* (Figure 35); *in* setae placed far from *e.i.p* margin; *Lam* present slightly higher up on margin, terminating anteriorly in large triangular *la.ti*, with *le* setae situated in the external limit of *la.ti* (Figures 30, 35) and the *l.l.f* terminating near internal limit of *la.ti* (Figure 26, 27). Insertions of *ro* and *le* setae at the same transverse level (Figure 34). The *l.l.f* is only clearly discernible under optical observation (Figures 26, 27); in SEM observation the zone between *l.l.f* and lamellar margin is a slightly flat zone (Figure 30). Laterally expanded spoon-shaped *Tu* appearing larger than *Pd I* (Figures 30, 35); very deep *s.tu.d* completely concealing leg I (Figure 30). Rounded, undulate rostral margin with prominent forward extension, parallel to *Tu*, extending backward *Pd I* level (Figures 30, 35) (See Discussion).

***Ventral region*** (Figures 34, 39, 40, 41, 42, 43). Large, clearly discernible rostral margin (Figure 34 indicated by X). Elevated epimera delimited by deep furrow (Figure 39); deep hollow zone paraxial to epimere 1(Figure 39, indicated by K); complete epimere *sj*; epimera 3 and 4 well discernible, unfused. Epimeral chaetotaxy 3-1-3-3. Epimeral setae *1a*, *2a*, *3a*, *4a*, largest. Discidum clearly discernible; *a.g.f* clearly visible, situated anterior to genital plate (Figure 40). Genital plate rounded, with four or five pairs of genital setae, (see Remarks) (Figures 40, 42); all setae more or less equal in length; *ag* setae situated posterior to genital opening, far from *ad_3_* (Figure 39). Three pairs of *ad* seta, more or less equal in length (Figure 41); anal plate polyhedral (Figure 41), sharply tipped; two pairs of anal setae; anterior pairs larger than posterior. Shallow depressions (*dep*) (Figure 39) situated laterally on either side of as well as between genital and anal openings. Subcapitulum diarthric (Figure 34); setae *h* largest.

***Legs*** (Figures 44–47). All legs monodactyle. Setal formulae I (1-4-2-4-16-1) (1-2-2); II (1-3-3-3-15-1) (1-1-2); III (2-3-1-2-14-1) (1-1-0); IV (1-2-2-3-12-1) (0-1-0). See Table 2.

**Table 2.** *Congocepheus kayoveae* sp. n. setae and solenidia.

Leg I	Femur	Genu	Tibia	Tarsus	Claw
setae	*da*, *dp*, *v*, *l*”	*d*,*v*	(*v*), *l*”,*d*	(*ft*),*ε*,(*tc*),(*it*),(*p*),(*u*),(*a*), *s*,(*pv*), *Ad*”	1
solenidia		*σ*	*φ_1_*, *φ_2_*	*ω _1_*_,_ *ω _2_*	
**Leg II**					
setae	*dp*, *da*, *l*’	*d*,*l*’,*v*	*v*, *d*, *l*’	(*pv*), *s*,(*a*),(*u*),(*p*), (*it*), (*tc*), (*ft*)	1
solenidia		σ	φ	ω _1_, ω _2_	
**Leg III**					
setae	*d*, *l*’,*v*	*l*’	(*v*)	(*pv*),*s*,(*a*),(*u*),(*p*),(*it*),(*tc*), *ft*’’	1
solenidia		σ	φ	-	
**Leg IV**					
setae	*d* , *v*	*d*, *l*’	*l*’, (*v*)	(*pv*), *s*,(*a*),(*u*), (*p*), (*tc*), *ft*”	1
solenidia		-	φ	-	

#### Remarks.

In some specimens the cerotegumental layer appears damaged (Figure 33), as more than 40 years have passed since collection, preservation of specimens may have been influenced by the quality of the initial diluted alcohol. Material of much greater age has been studied previously without problems, but in this case, the description of the cerotegumental layer must be regarded as provisional.

Twisting setae complicate and obscure observation; use of SEM vital in providing adequate information, while small particles adhering to setal surfaces further complicate observation. In several cases study material presents slight genital neotrichy; in two instances five pairs of setae were observed. One example of neotrichy was observed in *Congocepheus*, with notogastral neotrichy present in *Congocepheus germanicus*; but *Congocepheus kayoveae* is the first observed occurrence of genital neotrichy.

## Discussion

The two species described in this paper are related to *Congocepheus taurus* Balogh 1961. Unfortunately, as we were unable to obtain the type material, *Congocepheus taurus* is the only species within the genus *Congocepheus* we were unable to study. The type locality given by Balogh is “Africa Orientalis: Meru”, and searching through our material from Tanzania, we were unable to locate this species.

The description given by Balogh 1961 (page 522) is short and imprecise with only two figures, 10 (dorsal) and 11 (lateral) (page 523); figures lack detail, with important omissions. The following comparison is confined to an analysis of characters and figures provided by the author in 1961.

Commonalities: *Congocepheus taurus* and *Congocepheus rwandensis* are similar in terms of body shape; presence of irregular cord-shaped structures on notogaster; one central vein present on setae *c_1_* and *in*; *e.i.p* elevated with *in* setae situated anteriorly; *p.p.d* and *n.a.d* determine a large depression; presence of *f.l.p*. *Congocepheus taurus* is similar to *Congocepheus kayoveae* with regard to the presence of irregular cord-shaped structures on notogaster; *c_1_* setae with one central vein; presence of *f.l.p*.

Differences: *Congocepheus taurus* differs from *Congocepheus rwandensis* in terms of very short *c_1_* setae; *e.i.p* divided; *f.l.p* very different in shape; disposition, direction and shape of notogastral setae. *Congocepheus taurus* differs from *Congocepheus kayoveae* in terms of body shape; very short setae *c_1_*; *p.p.d* and *n.a.d* different in shape and size; greatly differing shape of *e.i.p*; *in* setae with only one vein; disposition and direction of notogastral setae; *f.l.p* very different shape.

The complexity of several structures present in species studied, necessitated observation from many different angles, as was the case in *Antongilibodes paulae* Fernandez et al., 2014 and *Mangabebodes kymatismosi* Fernandez et al., 2014. In the description of *Congocepheus kayoveae* sp. n., Figures 29, 30, 31, and 34 are complementary; lateral, frontal and frontal inclined views permit understanding of several characteristics and aspects not clearly observed (or difficult to interpret) in only the lateral or ventral position. Succinct studies such as that of *Congocepheus taurus*, with a short description and poorly developed figures, confound comparison, and several particularities of this species may go unnoticed.

In *Congocepheus kayoveae* sp. n. a similar situation was observed to that in *Mangabebodes kymatismosi*, Fernandez et al. 2014; where the *tutorium* forms a prominent lateral expansion and is relatively large; considered to be the first instance where this particularity is observed in *Congocepheus*. In *Congocepheus kayoveae* sp. n., the *s.tu.d* is very deep, completely concealing leg I.

Other interesting aspects are the position of the lateral setae (*l*”) of genu II (Figure 20), which during the leg folding process (See Fernandez et al. 2013a) protect the opening of the bothridium, and the perfect coaptation of the legs and depressions during leg folding (Figure 29).

## Supplementary Material

XML Treatment for Congocepheus rwandensis

XML Treatment for Congocepheus kayoveae
